# A Pilot Randomized Controlled Clinical Trial Comparing Piezo Versus Conventional Rotary Surgery for Removal of Impacted Mandibular Third Molars

**DOI:** 10.3390/bioengineering9070276

**Published:** 2022-06-25

**Authors:** Joana Saraiva Amaral, Carlos Miguel Marto, João Farias, Daniela Alves Pereira, Jorge Ermida, Álvaro Banaco, António Campos Felino, Francisco Caramelo, Sérgio Matos

**Affiliations:** 1 Dentistry Department, Institute of Oral Medicine and Surgery, Faculty of Medicine, University of Coimbra, 3000-075 Coimbra, Portugal; dpereira@fmed.uc.pt (D.A.P.); jorgermida@gmail.com (J.E.); alvarobanaco@hotmail.com (Á.B.); 2 Institute of Experimental Pathology, Faculty of Medicine, University of Coimbra, 3000-548 Coimbra, Portugal; cmiguel.marto@uc.pt; 3 Institute of Integrated Clinical Practice, Faculty of Medicine, University of Coimbra, 3000-075 Coimbra, Portugal; 4 Institute for Clinical and Biomedical Research (iCBR), Area of Environment, Genetics and Oncobiology (CIMAGO), Faculty of Medicine, University of Coimbra, 3000-548 Coimbra, Portugal; fcaramelo@fmed.uc.pt; 5 Centre for Innovative Biomedicine and Biotechnology (CIBB), University of Coimbra, 3000-548 Coimbra, Portugal; 6 Clinical and Academic Centre of Coimbra (CACC), 3004-561 Coimbra, Portugal; 7 Private Clinical Practice, CliFarias, 3810-157 Aveiro, Portugal; joao_farias@hotmail.com; 8 Centre for Innovation and Research in Oral Sciences (CIROS), Faculty of Medicine, University of Coimbra, 3000-075 Coimbra, Portugal; 9 Department of Oral Surgery and Periodontology, Faculty of Dental Medicine, University of Porto, 4200-135 Porto, Portugal; antonioccfelino@gmail.com; 10 Laboratory of Biostatistics and Medical Informatics, Faculty of Medicine, University of Coimbra, 3000-548 Coimbra, Portugal; 11 Dentistry Department, Institute of Periodontology, Faculty of Medicine, University of Coimbra, 3000-075 Coimbra, Portugal

**Keywords:** piezosurgery, piezoelectric, conventional rotary instruments, impacted third molars, pain, trismus, swelling

## Abstract

Background: The extraction of impacted mandibular third molars is a frequent dental surgery, interfering with patients’ quality of life. Ultrasonic surgery is an alternative to osteotomy with conventional rotary instruments. This study compares postoperative signals and symptoms after extracting impacted mandibular third molars using ultrasonic surgery or conventional rotary osteotomy. Methods: A pilot randomized controlled clinical trial was conducted. Thirty patients were randomly divided into the test group (ultrasonic technique) and a control group (conventional rotatory technique). All surgeries were timed. Swelling parameters, trismus and paraesthesia were evaluated on the day of surgery and the third, fifth and seventh postoperative days. Intraoperative bleeding was evaluated during surgery. Postoperative pain was evaluated daily by the patient through a visual analogue scale and the number of ingested analgesics. Results: Pain, swelling and trismus present beneficial results with the ultrasonic technique but without statistical significance. Intraoperative bleeding was significantly lower with ultrasonic surgery (t(28) = 3.258; *p* = 0.003). Operating time was significantly higher in extractions involving osteotomy and cutting crown and roots either with the conventional technique (*p* = 0.020) or ultrasonic technique (*p* = 0.039). Regardless of the surgical difficulty, no statistically significant results were detected between techniques regarding the procedure duration. Conclusions: The beneficial postoperative signs and symptoms make ultrasonic surgery a favourable therapeutic option, especially when the integrity of noble anatomical structures is the most important risk factor. Further studies with larger samples are needed to support the use of piezosurgery as a valid option for impacted mandibular third molar extraction.

## 1. Introduction

Extraction of mandibular third molars is one of the most common and delicate surgeries in dental practice [1,2,3,4,5]. According to the degree of inclusion, angulation, tooth location and bone density, the level of surgical difficulty will be reflected in the severity of signs and symptoms postoperatively [2,6,7,8]. Pain, swelling and trismus are common in this type of surgery [1,5,9,10,11,12]. Minimizing postoperative and not interfering with patients’ quality of life are the primary aims of the surgeon [6,13].

Osteotomy is one of the most critical and crucial steps in impacted mandibular third molar extraction. Many techniques are used for this, and if not used cautiously, they can be hazardous [2,8,14,15]. Thus, ultrasonic surgery arises as an alternative technique to conventional rotary osteotomy instruments [3,6]. The use of ultrasound was first proposed in the late 1970s by Horton et al. [2,16]. In 2004, Professor Tomaso Vercellotti developed a device which was directly applicable to oral and maxillofacial surgery [16,17]. Since then, its use has been extended to a wide range of surgical procedures, such as enucleation of cysts, osteogenesis distraction, expansion of bone crest, periodontal and endodontic surgery, maxillary sinus floor elevation, inferior alveolar nerve decompression, bone grafts, biopsies and dental extractions, especially of impacted teeth [3,6,16,18]. The device produces a modulated piezoelectric ultrasonic frequency from 24 to 29 kHz and micro-vibrations with an amplitude between 60 and 200 µm/s [1,19,20]. These characteristics provide a micrometric cutting ability, involving a minimum surface area, allowing a reduced manipulation of the soft tissues [6,21,22,23,24]. It presents precise cutting power with improved tactile control and without the need for high pressure, which is an advantage over rotary instruments [2,3,14,22,25]. Macro-vibrations associated with pressure induce a high tissue temperature, which is reflected in irregular cutting surfaces with margins of osteonecrosis [2,14]. Bone structure preservation after ultrasonic surgery allows a faster healing process, reflecting the levels of osteosynthesis [17,21,22,23].

The considerable advantage of the piezoelectric instrument is tissue selectivity, allowing cutting capacity only at the level of mineralized tissues [18,19,21,26,27]. In addition, it is an atraumatic technique, which ensures the integrity of mucosa, blood vessels and nerves, thus avoiding the occurrence of iatrogenic injury and decreasing postoperative levels of morbidity [3,6,21,23,26]. Another significant benefit of ultrasonic surgery is intraoperative visibility, resulting from the cavitation phenomenon [16,22,23]. Instrument irrigation in symbiosis with micro-vibrations promotes gas bullae into the lumen of blood vessels during bone cutting, causing a haemostasis effect and reducing blood loss [6,16,26,28,29].

Since 2004, ultrasonic surgery has been considered safe, efficient, and a beneficial technique for patients regarding postoperative symptoms [19,22,26,30]. However, surgical time is still a persistent and limiting factor to its applicability [3,18,26]. Meanwhile, oral and maxillofacial surgeons believe in overcoming this disadvantage with experience [22,28].

The main goal of this randomized controlled study is to compare pain in impacted mandibular third molar extraction using two surgical techniques: ultrasonic osteotomy surgery and conventional rotatory osteotomy. Secondly, we intend to evaluate the impact of the surgical difficulty and duration of the procedure with postoperative signs and symptoms. The results of the two techniques relative to postoperative swelling, trismus, paraesthesia, and intraoperative bleeding will also be compared.

## 2. Materials and Methods

### 2.1. Study Design

A pilot randomized controlled clinical trial with a parallel design was conducted which included 30 patients, equally and randomly distributed in the test group (*n* = 15) or the control group (*n* = 15). Each patient received only one of the procedures and was followed longitudinally on the third, fifth and seventh days.

Randomization was established with computer software by generating random sequential numbers placed in opaque envelopes. The treatment allocation was performed after the surgical incision and the respective detachment of the flap for bone exposure of the area to be operated on. After opening the sealed envelopes, the operator was informed of the designated treatment.

The surgeries and measurements of the postoperative controls were performed by the same properly calibrated operator (J.S.A.).

The study respected the principles of the Helsinki Declaration for clinical research in humans. All patients were informed of the surgical protocol, follow-up periods and possible risks and complications inherent to the procedure and signed an informed consent form. The experimental protocol and the respective informed consent were approved by the Ethics Committee of the Faculty of Medicine, University of Coimbra (027-CE-2015).

This clinical trial was implemented and described following the Consolidated Standards of Reporting Trials (CONSORT) statement (http://www.consort-statement.org/ (accessed on 14 March 2022)). The CONSORT checklist is available as Supplementary Material S1. 

### 2.2. Patient Selection

The sample consisted of 30 patients recruited from the oral surgery consultation at the university clinic, dentistry area of the Faculty of Medicine, University of Coimbra.

Inclusion criteria were defined as patients with an impacted mandibular third molar, the extraction of which necessarily involved osteotomy, and in which the adjacent second molar was present: ASA I patients (without physiological or organic dysfunction).

All individuals with periodontal disease or recent episodes of pericoronitis, smokers, pregnant women or infants were excluded. In addition, chronic medication, allergy to penicillin or any other drug present in the defined protocol were also considered eliminatory factors.

### 2.3. Surgical Procedure

The sample was randomly divided into two groups: the test group in which the osteotomy was done using the piezosurgery technique and the control group, where the osteotomy was performed with the conventional rotary handpiece technique. The same operator performed all extractions, and their duration was timed, from the first incision to the last suture point.

Local anaesthesia with vestibular and lingual infiltration (4% articaine with 1: 100,000 epinephrine) and an inferior alveolar nerve block (3% mepivacaine without vasoconstrictor) were used.

The incision was made with a No. 15 scalpel blade, intra-sulcular from the mesial of the first mandibular molar to the distal of the second mandibular molar with a discharge incision along the mandible ascending branch, to raise a full-thickness flap and expose the cortical bone.

In both groups, when the crown or root section was required, a turbine laminated cylindrical tungsten bit (Bora L-BIEN AIR, Ref. 1600382-001) was used.

In the test group, osteotomy was performed with an NSK—Vario Surg 3 ultrasound system (model: VS3-LED-HPSC, reference: E1133), whose frequency varies between 28 and 32 kHz. The H SG1 tip was used since it has a greater cutting capacity, allowing the use of the maximum power of the instrument, which is increased 50% in relation to previous models.

In the control group, osteotomy was performed with a spherical laminated tungsten bit mounted on a handpiece (Ref.1600383-001BIEN AIR).

The socket was irrigated with a saline solution, and the flap was repositioned and sutured with a non-absorbable 5-0 polyamide monofilament thread (Seralon^®^ 5-0).

The post-surgical protocol included local haemostatic measurements, with the placement of a compress for 30 min and the application of ice for 15 min every hour for the six hours following surgery.

Amoxicillin and clavulanic acid tablets 875/125 mg 16 12/12 h, ibuprofen 600 mg 12/12 h for three days and paracetamol 1000 mg in the SOS regimen were prescribed.

Patients were instructed to rinse with 0.2% chlorhexidine three times a day for seven days. The suture was removed on the seventh postoperative day.

### 2.4. Evaluation Parameters

#### 2.4.1. Surgical Difficulty

The surgical difficulty was assessed according to anatomical characteristics, through radiographic analysis, based on the classification by Pell and Gregory (1933), which includes the depth of inclusion and the relationship with the ascending branch of the mandible; and Winter’s classification (1926), which is based on the angulation of the third molar long axis relative to that of the second molar [31,32].

In addition to this preoperative classification, intraoperative factors were also considered, namely the surgical manoeuvres necessary for extraction, using a modified version of the Parant scale (1974), which is divided into four stages: extraction only with forceps (I), extraction with an osteotomy (II), extraction with osteotomy and crown section (III) and extraction with osteotomy and crown and roots section (IV) [33]. However, stage I was eliminated from the study, as one of the inclusion criteria was the need for osteotomy.

#### 2.4.2. Postoperative Pain

The pain was assessed using a visual analogue scale (VAS) numbered from 0 to 10, corresponding to the minimum value for the absence of pain and the maximum value for the most severe pain [34]. Patients were instructed to quantify the pain immediately before surgery, corresponding to zero and then daily at about the same time until the seventh postoperative day.

This variable was also assessed by the number of analgesics (paracetamol 1000 mg) taken daily by patients in both groups.

#### 2.4.3. Swelling

The swelling was assessed using two facial measurements, using a millimetre tape from the tragus (Tg) to the labial commissure (LC) and from the tragus to the cutaneous chin/ment (CM) [7,35]. A reference measurement was made immediately before surgery, with the remaining measurements being made on the third, fifth, and seventh postoperative days. The difference between these measurements corresponded to the swelling caused by the surgery.

#### 2.4.4. Trismus

To assess post-surgical trismus, a first reference measurement of the maximum mouth opening was performed, with a calliper, from the incisal edge of 11 to the incisal edge of 41, immediately before surgery [35]. This measurement was repeated on the control days (third, fifth and seventh postoperative days). The difference between the first and subsequent measurements reproduced the limitation of the mouth opening.

#### 2.4.5. Paraesthesia

Paraesthesia or hypoesthesia was assessed by the nociceptive needle pressure test. With a sterile needle, superficial pricks were made directly on the patient’s skin, at the lower lip level and the mental region of the intervention side [36]. The affected area was mapped and photographed.

#### 2.4.6. Operative Bleeding

This was evaluated independently by the operator and the assistant using a VAS, numbered from 0 to 10, where 0 corresponds to the minimum of bleeding and 10 to the maximum of bleeding [34].

### 2.5. Statistical Analysis

This study was designed as a pilot equivalence trial between the two techniques. The pain variable was considered the main outcome for the sample size calculation.

The collected data were objects of description and statistical interpretation using appropriate numerical indicators.

The characterization of the sample regarding gender, age and analysis of its homogeneity between the test and control groups was obtained through Student’s t-test for independent samples and Fisher’s exact test.

According to the pre- and intraoperative classifications adopted in both groups, the surgical difficulty was described based on histograms, comparing the medians obtained by the Mann–Whitney test.

The analysis of the operating time as a function of the surgical difficulty in both groups (test and control) was obtained by the chi-square test by Monte Carlo simulation. The same test was used to assess the duration of surgery according to the surgical technique adopted.

Postoperative signs and symptoms (pain, swelling and trismus) and operative bleeding were described by graphs and tables, being evaluated by the ANOVA test of repeated measures. The paraesthesia parameter was not subject to any interpretation since it was not found in any case, nor were there any other intra and postoperative complications.

The statistical analysis was performed using the IBM^®^ SPSS^®^ v20 statistical calculation program.

## 3. Results

**Figure 1:**
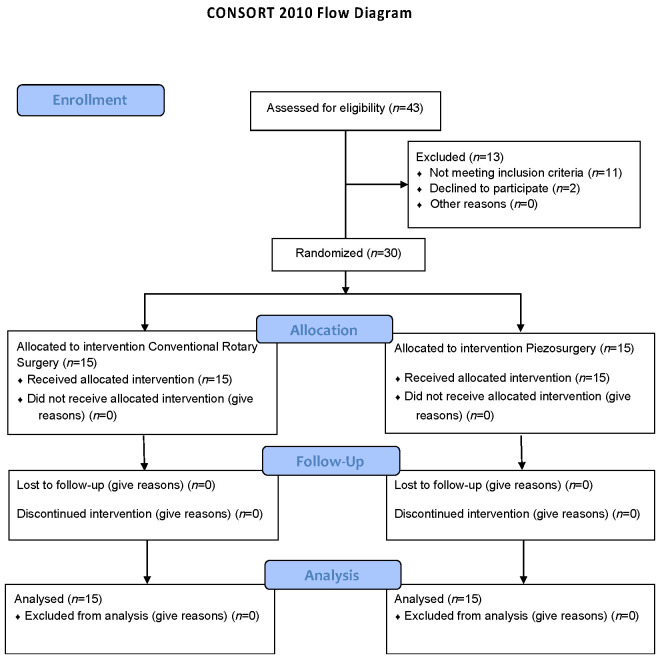
PRISMA flow chart.

The PRISMA flow chart of this study is presented in Figure 1.

### 3.1. Characterization of the Sample According to Age and Gender

The sample consisted of 7 males, 3 in the control group and 4 in the test group and 23 females, 12 belonging to the control group and 11 to the test group. The mean age of the participants in the control group was 27 ± 6 years and in the test group 26 ± 6 years. No statistical differences existed between the two groups.

### 3.2. Characterization of the Sample According to the Surgical Difficulty

To determine the surgical difficulty, preoperative parameters (Pell and Gregory and Winter’s classification) and intraoperative parameters (modified version of the Parant scale) were considered.

#### 3.2.1. Pell and Gregory Classification

**Figure 2:**
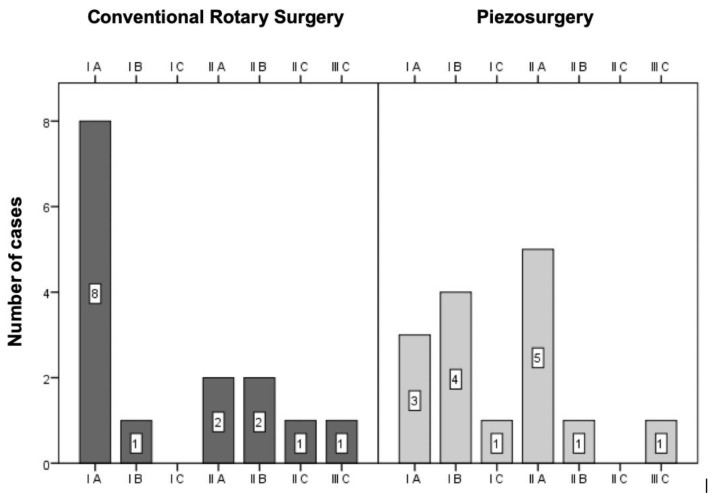
Sample distribution according to the Pell and Gregory classification (*p* = 0.247 for the comparison between the two techniques).

Of the 30 impacted mandibular molars included in the sample, eleven were in position IA, five in position IB, one in position IC, seven in position IIA, three in position IIB, one in position IIC and two in position IIIC (Figure 2).

The distribution of cases between the two groups was assessed using the chi-square test by Monte Carlo simulation, with no statistically significant differences (*p* = 0.247).

#### 3.2.2. Winter’s Classification

**Figure 3:**
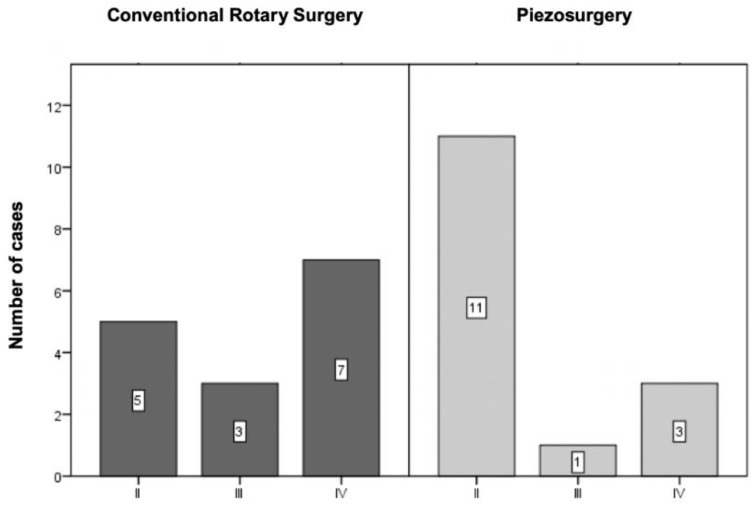
Sample distribution according to Winter’s classification (*p* = 0.397 for the comparison between the two techniques).

According to Winter’s classification, half of the sample was formed by vertical third molars. Of the remaining fifteen, five were mesioangular, six were horizontal, and four were distoangular (Figure 3).

The distribution of cases between the two groups was assessed using the chi-square test by Monte Carlo simulation, with no statistically significant differences (*p* = 0.397).

#### 3.2.3. Modified Version of the Parant Scale

**Figure 4:**
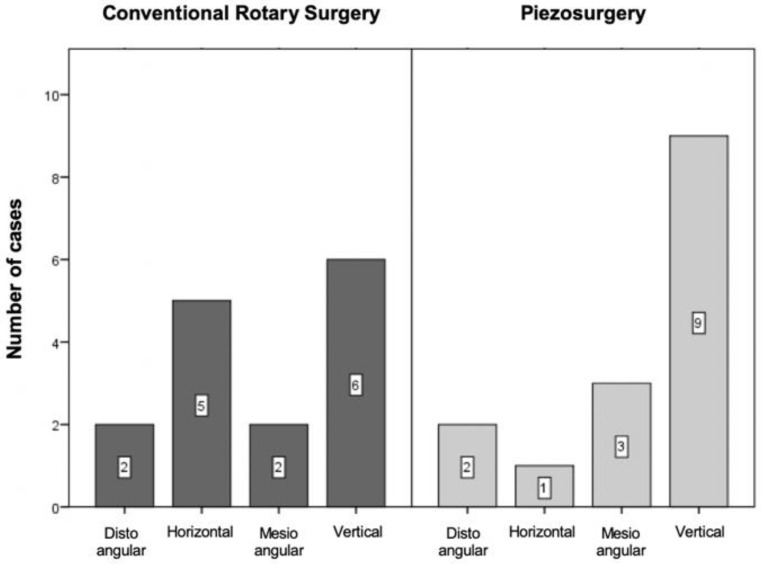
Sample distribution according to the modified version of the Parant scale (*p* = 0.136 for the comparison between the two techniques).

Analysing the operative difficulty through the required surgical manoeuvres, it was found that in the control group, two-thirds of the extractions involved odontectomy (three—of the crown, seven—odontectomy of the crown and roots) and one-third only osteotomy (Figure 4. In contrast, in the test group, most extractions involved only osteotomy, presenting only one case in which odontectomy of the crown was required and three cases in which odontectomy of the crown and roots was necessary (Figure 4).

The distribution of cases between the two groups was assessed using the chi-square test by Monte Carlo simulation, with a *p* = 0.136 value, with no significant differences.

### 3.3. Operating Time vs. Surgical Difficulty vs. Surgical Technique

The analysis of the operating time according to the surgical difficulty and the applied technique was obtained by the chi-square test by Monte Carlo simulation.

#### 3.3.1. Pell and Gregory Classification

According to the Pell and Gregory classification, it was found that when the conventional technique was applied, the surgical difficulty in no way interfered with the operating time (CC = −0.241, *p* = 0.388).

**Figure 5:**
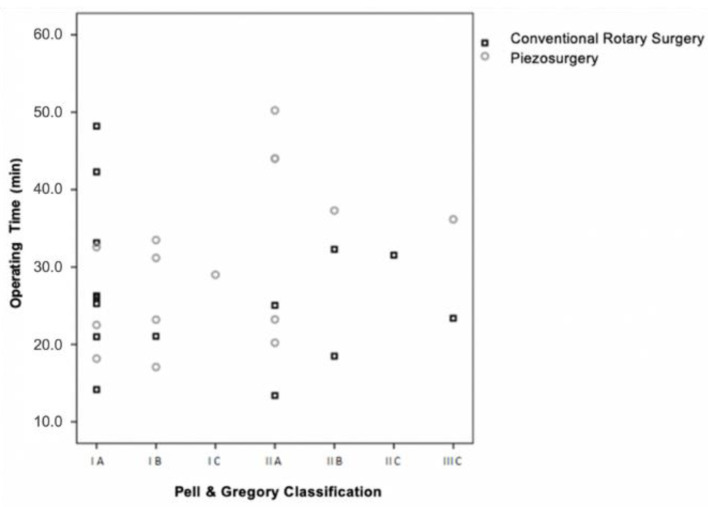
Operating time vs. surgical difficulty (Pell and Gregory) vs. surgical technique (*p* = 0.388 for the conventional rotary surgery and *p* = 0.030 for the piezosurgery).

On the contrary, in the ultrasonic technique, there was a moderate correlation between the surgical duration and the degree of difficulty (CC = 0.560, *p* = 0.030) (Figure 5).

#### 3.3.2. Winter’s Classification

**Figure 6:**
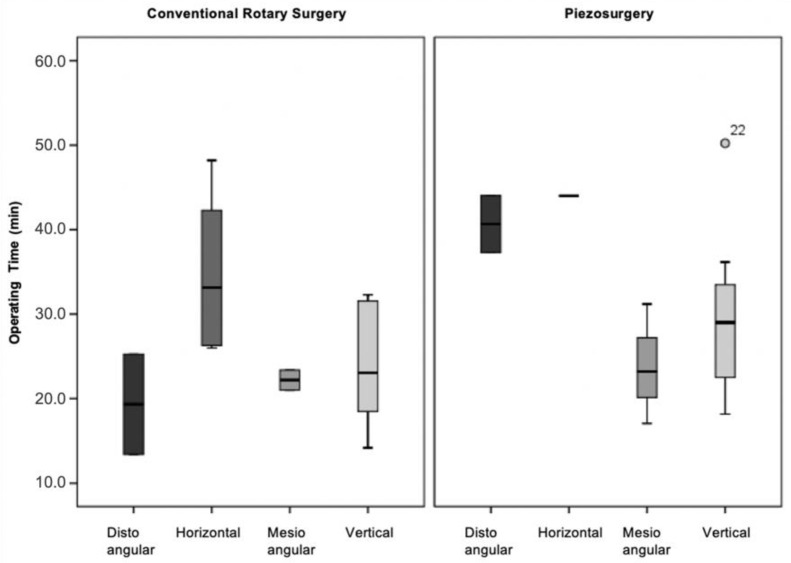
Operative time vs. surgical difficulty (Winter’s classification) vs. surgical technique (*p* = 0.070 for the conventional rotary surgery and *p* = 0.135 for the piezosurgery).

According to Winter’s classification, there were no statistically significant differences in operating time in the four classes of surgical difficulty (distoangular, horizontal, mesioangular and vertical) in either the conventional technique ($\chi^{2}\left(3\right)=7.068;p=0.070$), or in the ultrasonic technique ($\chi^{2}\left(3\right)=5.556;p=0.135$) (Figure 6).

#### 3.3.3. Modified Version of the Parant Scale

**Figure 7:**
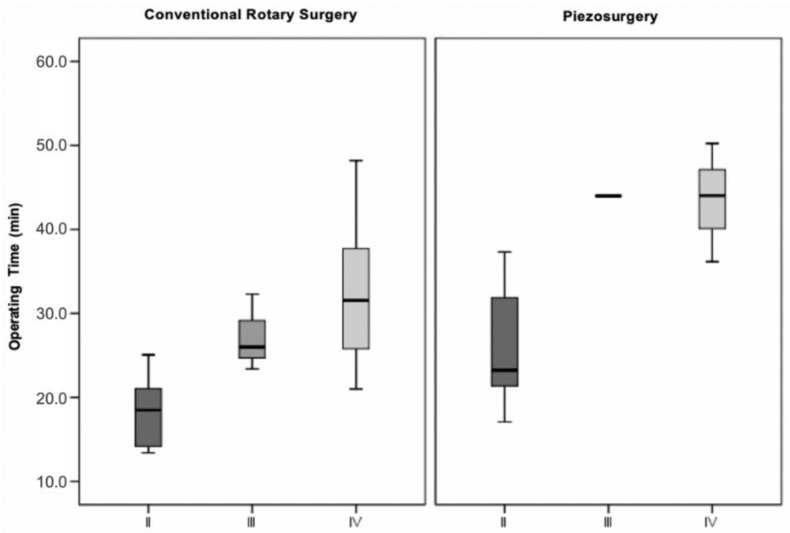
Operative time vs. surgical difficulty (modified version of the Parant scale) vs. surgical technique (*p* = 0.023 for the conventional rotary surgery and *p* = 0.023 for the piezosurgery).

Defining the surgical difficulty by intraoperative manoeuvres, it was found that in extractions in which odontectomy of the crown and roots was necessary (stage IV), the surgical time was significantly longer compared to extractions in which only osteotomy was required (stage II). This statistically significant difference in operating time was found both in the conventional technique ($\chi^{2}\left(2\right)=7.569;p=0.023$), and in the ultrasonic technique ($\chi^{2}\left(2\right)=7.521;p=0.023$) between stages II and IV (conventional technique *p* = 0.020; ultrasonic technique *p* = 0.039) (Figure 7).

### 3.4. Operating Time vs. Surgical Technique

**Figure 8:**
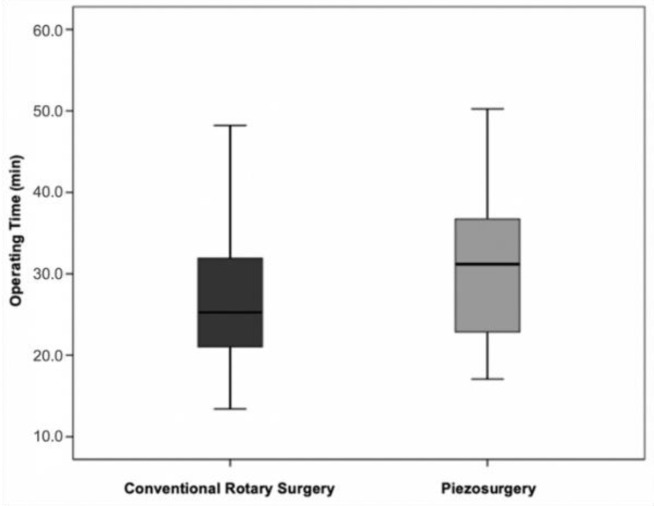
Operative time vs. surgical technique (*p* = 0.271 for the comparison between the two techniques).

Analysing the operating time in the two surgical techniques, regardless of the surgical difficulty, it was found that the ultrasonic technique had an average duration of 30.8 min (SD = 9.6) and the conventional rotary technique an average duration of 26.8 min (SD = 9.9). This difference between techniques of about four minutes was not statistically significant (t(28)=−1.12;p=0.271) (Figure 8).

### 3.5. Postoperative Pain

#### 3.5.1. Pain vs. Surgical Technique (VAS)

The pain was evaluated daily by the patient. Analysing the mean values (± standard deviation) of pain for the two operative techniques, the same values were found in six of the eight days. Different pain levels were registered on the first postoperative day, in which conventional surgery had higher values and on the seventh postoperative day, in which ultrasound surgery showed higher values. The differences between the control and test groups were not statistically significant (F(7; 196)=0.334;p=0.938) (Table 1).

#### 3.5.2. Pain vs. Surgical Technique (Number of Analgesics)

There was a smaller number of analgesics in the group corresponding to the ultrasonic technique, except for day 0 when the average value of analgesics was equal to two groups. Even so, the differences were not statistically significant (F(1, 28)=30.723; p=0.511) (Table 1).

### 3.6. Swelling

#### 3.6.1. Swelling vs. Surgical Technique (Tg-LC)

The swelling was evaluated on the third, fifth and seventh days. Analysing the mean values with the standard deviation of the swelling, lower values were found on the third and fifth postoperative days in the ultrasonic technique. In contrast to the seventh postoperative day, this surgical technique showed, tangentially, a worse result. Even so, the differences obtained were not statistically significant (F(1.25; 56)=2.335;p=0.130) (Table 2).

#### 3.6.2. Swelling vs. Surgical Technique (Tg-CM)

Evaluating the swelling by the Tg-CM facial measurement, the results proved to be more favourable in the ultrasonic technique during the three days of postoperative control, yet the differences were not statistically significant (F(2; 56)=1.278;p=0.287) (Table 2).

### 3.7. Trismus vs. Surgical Technique

Trismus was evaluated on the third, fifth and seventh days. On the third day, the patients in the test group showed a greater limitation of the mouth opening, which was reversed on the fifth and seventh days. Thus, ultrasound surgery showed better results, except for the third postoperative day. Even so, the differences between the two techniques were not statistically significant (F(1.47; 56)=0.82;p=0.765) (Table 3).

### 3.8. Operative Bleeding vs. Surgical Technique

**Figure 9:**
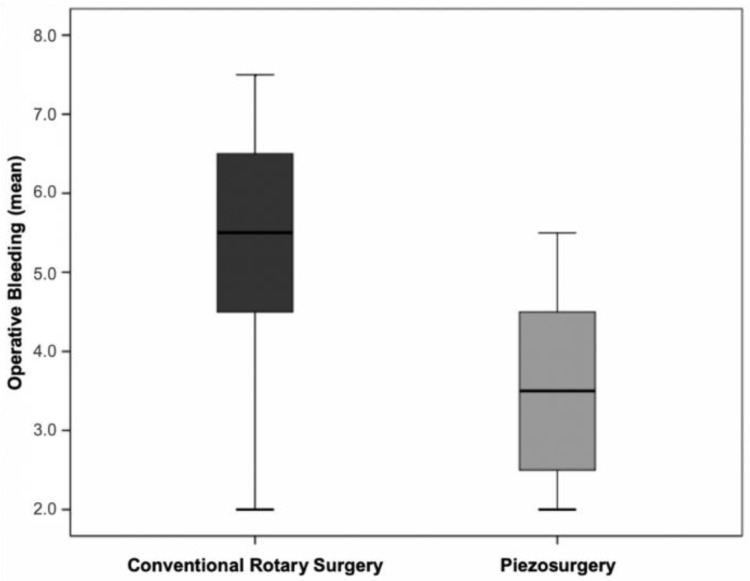
Distribution of the levels of operative bleeding in the two surgical techniques (*p* = 0.003 for the comparison between the two techniques).

Analysing the average bleeding values in the two surgical techniques, higher values were obtained in the conventional technique ($\bar{x}=5.33;s=1.71$), compared to the ultrasonic technique ($\bar{x}=3.60;s=1.15$). These differences were statistically significant (t(28)=3.58;p=0.003) (Figure 9).

The patients reported no complications during the follow-up period.

## 4. Discussion

Surgical planning in symbiosis with provision for operative difficulty significantly reduces the risk of intraoperative complications [6,23]. To satisfy this premise and to ensure high surgical efficacy with less physical and psychological trauma for the patient, alternative techniques to conventional rotational surgery have emerged in recent years, with an emphasis on ultrasonic surgery [3,26].

In the present clinical trial, the two surgical techniques in the extraction of impacted mandibular third molars were compared, trying to determine the extent to which the degree of surgical difficulty and the osteotomy technique interfere in the surgical duration and the severity of the postoperative signs and symptoms.

According to the Pell and Gregory classification, there was a moderate correlation between operating time and the degree of surgical difficulty for the ultrasonic technique. However, such a correlation was not evident in conventional surgery.

According to the Winter classification, no statistically significant differences existed between the operating time and the degree of surgical difficulty in any technique. Comparing the results with those in the literature, a study by Mantovani et al. [22], used the same preoperative parameters to assess the surgical difficulty and found that the operating time did not depend on the degree of inclusion or the angle of the third mandibular molars in both surgical techniques. This discrepancy in results may be due to the number of operators since in the study by Mantovani et al. [22], the surgeries were performed by three surgeons with different clinical experiences, whereas in the present clinical trial, all surgeries were performed by the same operator with less clinical experience in the ultrasonic technique [22].

Using the modified version of the Parant scale, which is guided by intraoperative manoeuvres, it was found in both surgical techniques that the extractions that involved only osteotomy (stage II) took significantly less time than the extractions that required osteotomy and odontosection of the crown and roots (stage IV). According to Rullo et al. [17], this statistical significance was only denoted in the ultrasonic technique; however, in their interpretation of results they did not distinguish stage III (crown section) from stage IV (crown and root section), which may justify the difference in results obtained [17].

Interpreting the operating time in isolation in the two surgical techniques, it was found that the duration of extraction of mandibular third molars included was longer in the ultrasonic technique. This result is corroborated by the meta-analyses by Cicciù et al. [26], Liu et al. [37], Al-Moraissi et al. [38] and Jiang et al. [39], obtaining a difference of 4.6 min between the last two techniques, which is close to the 4 min obtained in the present clinical trial. However, it should be noted that the differences obtained did not have statistical significance, contrary to what was presented in the meta-analyses mentioned above.

Concerning the pain factor, despite an observed trend towards more favourable results in ultrasonic surgery, there were no statistically significant differences. This might be due to the small sample size. The meta-analyses by Cicciù et al. [26], Liu J. et al. [37], Al-Moraissi et al. [38], Badenoch-Jones et al. [40] and Jiang et al. [39] support the results obtained, the first four presenting statistical significance.

Analysing the results separately according to the two measurement methods, with VAS, there was a beneficial difference in favour of the ultrasonic technique on the first postoperative day, in line with what was reported in the literature by Mantovani et al. 2014 [22]. This comfort in the immediate post-surgical period (day 1) may be inherent to the characteristics of the piezoelectric instrument. Its micrometric cutting capacity, associated with high tactile control, contrasts with the macro-vibrations and high pressure of the rotary instruments, which the patient perceives as discomfort and/or pain, resulting in higher anxiety levels [15,38,39]. In addition to this characteristic, the tissue selectivity of ultrasonic surgery is highlighted, which produces lower inflammatory levels and, therefore, lower levels of pain [15,38,39].

According to the number of analgesics ingested, the most favourable result of the ultrasonic technique was transversal to the entire postoperative period, except for the day of surgery when the pain levels were the same in both techniques. The same result, but imbued with statistical significance, was obtained in the studies by Goyal et al. (2012) and Barone et al. (2010), which in terms of sample size and study design, are homologous to the present clinical trial [6,28].

It is important to note that the measurement of the pain variable is very subjective and depends on biological factors such as the pain threshold, which is specific to each individual, and the age factor [38,39]. Age reflects inflammatory levels and neurological complications [21]. Bruce et al. (1980), cited by Piersanti et al. [21] consider that patients over the age of 35 years are predisposed to develop more aggravated postoperative conditions, with higher levels of pain, swelling and trismus. As age increases, there is a reduction in the thickness of the periodontal ligament associated with an increase in mineralization of the mandibular bone, making extractions more complex and traumatic [21]. According to Chiapasco et al. (1995), cited in the same study, these differences are already evident from the age of 24 [21].

The swelling was assessed on the third, fifth, and seventh postoperative days using two facial measurements considering three reference points (Tg; LC; CM). The results obtained were favourable for ultrasonic surgery, except for the seventh postoperative day, on which, according to the Tg-LC measurement, the swelling was greater in this technique.

According to Mantovani et al. [22], swelling reflects the intensity of tissue trauma and inflammatory process and presents a gradual evolution, reaching its peak at 48 h post-surgery [22]. In this sense, at least four [21,22,29,41] of the eight [6,21,22,28,29,41,42,43] studies included in the meta-analyses by Al-Moraissi et al. [38] and Jiang et al. [39] do not fully reflect the behaviour of this variable throughout the postoperative period, since they perform a single measurement on the seventh day, in which the swelling is already residual or even non-existent, or at 24 postoperative hours where the peak has not yet been reached. In addition to this factor, the heterogeneity of measurement methods is highlighted, making it difficult to compare and interpret the results.

Despite these biases, it is transversal to all studies that ultrasonic surgery induces less swelling than conventional rotary technique, with the meta-analyses by Liu J. et al. [37], Al-Moraissi et al. [38], Badenoch-Jones [40] and Jiang et al. [39] presenting statistically significant differences between techniques. The evidence available in the literature supports the results obtained in the present clinical trial, except for the seventh postoperative day, when higher swelling values were obtained in the ultrasonic technique according to the Tg-LC measurement. However, in the Tg-CM measurement, the result was already more favourable for ultrasound surgery, which leads us to believe that this specific discrepancy will not have any relevance or clinical reproducibility.

The lower swelling caused by the ultrasonic technique can be explained by the characteristics of the piezoelectric system itself, namely tissue selectivity, the micrometric cutting capacity and the cavitation effect, which results in less surgical trauma and, therefore, in lower levels of inflammation, bleeding and swelling [21,22,23,29,43].

Trismus was evaluated on the third, fifth, and seventh postoperative days, with a lower limit of mouth opening in the group where the ultrasonic technique was applied on the fifth and seventh days. On the third day, the trend was reversed, with conventional surgery showing better results. These differences had no statistical significance. These results were in agreement with those revealed in the meta-analyses by Al-Moraissi et al. [38], in which the differences were statistically significant, Liu et al. [37] and Jiang et al. [39], except for the third postoperative day and Badenoch-Jones et al. [40], except for the seventh postoperative day [37,38,39,40]. However, Barone et al. [6] presented a homologous result except for the fifth postoperative day. Such discrepancies may be related to a range of factors that influence the intensity of the inflammatory response, such as the patient’s age, biological diversity, or the degree of surgical difficulty. Cicciù et al. [26] found a statistically significant pain reduction on day 1 using piezosurgery, but such a time-point was not evaluated in the present study, so comparisons cannot be made.

According to Oikarinen, cited by Goyal et al. [28], the duration of a surgical procedure significantly influences postoperative pain and trismus levels [28]. However, Benediktsdottir et al. (2004), cited in the same study, contradict this assumption, stating that the intensity of signs and symptoms after surgery are independent of the operating time [28]. In fact, ultrasound surgery has a longer operating time than conventional surgery. Still, the limitation of the mouth opening and levels of pain and swelling are lower, which gives a more favourable postoperative to this surgical technique [8,15,20,37,38,39,40,44,45]. This may be due to its advantages over rotational surgery, which overcome the possible disadvantage of the time factor [6,22].

In the present clinical trial, operative bleeding was assessed by the operator and the assistant independently, using a VAS numbered 0–10. The results demonstrated that the ultrasonic technique induced significantly lower bleeding levels than the conventional technique. Sivolella et al. [29] used the same measurement method to assess this variable and obtained the same result without statistical significance. The lesser bleeding in ultrasound surgery may be due to the cavitation effect and the micrometric cutting capacity, making this technique less traumatic and reflecting a lesser severity of postoperative signs and symptoms [6,8,22,29]. Additionally, it facilitates the surgical procedure, improving operative visibility [6,22,29].

In choosing a specific surgical technique, maintaining physical integrity is an imperative factor, minimizing iatrogenic accidents and the quality of the postoperative period as much as possible. Of the three parameters evaluated—pain, swelling, and trismus—the results were beneficial in ultrasonic surgery. Despite the average difference between techniques being only four minutes, this value may be masked by the heterogeneity of the groups with disparate surgical difficulties, so the homogeneity of the samples is considered necessary in future clinical trials.

Regarding intraoperative complications, it is essential to emphasize tissue selectivity and the micrometric cutting capacity of ultrasonic surgery, which in extreme situations (proximity to noble anatomical structures) outweighs the disadvantage of the operating time. In the remaining cases, the use of this technique is an ambiguous decision. If, on the one hand, the technique endows postoperative results that tend to be more advantageous, on the other hand, the longer operating time can diminish this advantage. Thus, according to Piersanti et al. [21], in young patients, the technique of choice will remain conventional rotational surgery; given the histological characteristics of bone tissue—less mineralized and the greater thickness of the periodontal ligament—the preponderant factor in the quality of the postoperative period will be the shorter surgical time [21]. According to Chiapasco et al. (1995), cited by Piersanti et al. [21], in patients of older age groups, more than 24 years of age, in which bone tissue is already more mineralized and the periodontal ligament narrower, the primary objective will be to make extraction the least traumatic possible, assuming that the more extensive operating time is less relevant, and thus the technique of choice will be ultrasonic surgery. In addition to this dichotomy, the high cost of the equipment is highlighted. In this context, a cost-benefit analysis would be interesting to undertake in future studies.

The present study has a small sample size, so caution should be taken regarding the obtained conclusions. Due to the use of pain as the main outcome, which has a subjective measurement, the results can present bias related to reading and interpretation of the VAS. In future studies, we suggest changing the primary outcome to an objective mensurable variable such as swelling, which has a similar impact on the postoperative characterization. Additionally, since the relationship between age and swelling is not fully understood, further studies should include different age groups to clarify this. Patient report outcomes, including psychological aspects, can also be included in the evaluated parameters.

Additionally, the study design can be changed from an equivalence study to a superiority one. The obtained results were fundamental to calculating the sample needed for further studies. This way, considering a significance level of 5% and a 90% power, the enrolment of 62 patients is needed.

## 5. Conclusions

Piezosurgery tends to be beneficial in terms of postoperative signs and symptoms (pain, swelling, trismus), compared to conventional surgery. Although these differences are not statistically significant, they are clinically relevant since they translate into a more favourable postoperative period, interfering less with the patients’ quality of life. Additionally, it is a technique that, due to its lower noise and vibration, is associated with lower levels of anxiety and discomfort. Operative bleeding was also significantly less in this technique, given the less invasive surgical intervention, representing an advantage for the patient.

Despite the technical demands and high equipment costs, the inherent advantages of the technique make its clinical applicability beneficial, especially in cases where the integrity of the noble anatomical structures is the most relevant risk factor.

## Figures and Tables

**Table 1:** Quantification of pain (VAS scale and number of analgesics) according to the operative technique throughout the post-surgical days.

Post-Surgical Days	Conventional Rotary Surgery		Piezosurgery	
	VAS	Analgesics Number	VAS	Analgesics Number
Day 0	4 ± 3	0.87	4 ± 3	0.87
Day 1	5 ± 3	1.07	4 ± 3	0.87
Day 2	4 ± 2	0.93	4 ± 3	0.80
Day 3	3 ± 2	0.73	3 ± 2	0.40
Day 4	2 ± 2	0.47	2 ± 2	0.40
Day 5	2 ± 1	0.33	2 ± 1	0.20
Day 6	1 ± 1	0.2	1 ± 1	0.13
Day 7	0 ± 1	0.07	1 ± 1	0.00

**Table 2:** Quantification of swelling (Tg-LC and Tg-CM) on the third, fifth and seventh postoperative days in both surgical techniques.

Post-Surgical Days	Conventional Rotary Surgery		Piezosurgery	
	Tg-LC	Tg-CM	Tg-LC	Tg-CM
Day 3	6.9 ± 6.2	7.3 ± 3.4	4.1 ± 2.2	5.6 ± 4.2
Day 5	4.5 ± 2.6	5.0 ± 3.5	2.7 ± 2.3	3.5 ± 4.1
Day 7	0.7 ± 1.1	0.7 ± 1.6	0.8 ± 1.6	0.6 ± 1.8

**Table 3:** Quantification of trismus on the third, fifth and seventh postoperative days in both surgical techniques.

Post-Surgical Days	Conventional Rotary Surgery	Piezosurgery
Day 3	−14.5 ± 8.7	−15.1 ± 8.7
Day 5	−10.1 ± 7.5	−9.5 ± 7.1
Day 7	−4.3 ± 5.5	−4.1 ± 6.0

## Data Availability

The data presented in this study are available on request from the corresponding author.

## Supplementary Materials

The following supporting information can be downloaded at: https://www.mdpi.com/article/10.3390/bioengineering9070276/s1, Figure S1: CONSORT checklist.
